# Supplementary material for: MetaQTL: a package of new computational methods for the meta-analysis of QTL mapping experiments
Source: BMC Bioinformatics. 2007 Feb 8;8:49. doi: 10.1186/1471-2105-8-49 (PMC1808479; doi:10.1186/1471-2105-8-49)
Supplement: Additional File 7 — MetaQTL Package : jar file and tutorial. This Zip archive contains both the MetaQTL JAR file and the files of the tutorial. [file 1471-2105-8-49-S7.zip › org.inra.metaqtl/doc/org/thalia/bio/IBioIndividual.html]

IBioIndividual


|  |  |  |
| --- | --- | --- |
| **Overview** \| **Package** \| **Class** \| **Use** \| **Tree** \| **Deprecated** \| **Index** \| **Help** | |  |
| **PREV CLASS**   **NEXT CLASS** | **FRAMES**    **NO FRAMES**     **All Classes** |  |
| SUMMARY: NESTED \| FIELD \| CONSTR \| METHOD | DETAIL: FIELD \| CONSTR \| METHOD |  |


---


## org.thalia.bio Interface IBioIndividual

**All Superinterfaces:**: IBioAdaptable, IBioEntity

---

``` public interface IBioIndividual extends IBioEntity ```

Class Description Here

**Author:**
:   Jean-Baptiste Veyrieras

---

| **Method Summary** | |
| --- | --- |
| `IBioIndividual[]` | `getParents()`             Returns the pedigree of the individual |
| `IBioPopulation` | `getPopulation()` |
| `void` | `setPopulation(IBioPopulation population)` |

**Methods inherited from interface org.thalia.bio.IBioEntity**

`getName, getParent, getProperties, getType, setName, setProperties`

**Methods inherited from interface org.thalia.bio.IBioAdaptable**

`getBioAdapter`

**Method Detail**

### getParents

```
IBioIndividual[] getParents()
```

:   Returns the pedigree of the individual

---


### getPopulation

```
IBioPopulation getPopulation()
```

---


### setPopulation

```
void setPopulation(IBioPopulation population)
```


---


|  |  |  |
| --- | --- | --- |
| **Overview** \| **Package** \| **Class** \| **Use** \| **Tree** \| **Deprecated** \| **Index** \| **Help** | |  |
| **PREV CLASS**   **NEXT CLASS** | **FRAMES**    **NO FRAMES**     **All Classes** |  |
| SUMMARY: NESTED \| FIELD \| CONSTR \| METHOD | DETAIL: FIELD \| CONSTR \| METHOD |  |


---
